# Predictive biomarkers for anti-TNF alpha therapy in IBD patients

**DOI:** 10.1186/s12967-024-05058-1

**Published:** 2024-03-16

**Authors:** Manoj Kumar, Selvasankar Murugesan, Nazira Ibrahim, Mamoun Elawad, Souhaila Al Khodor

**Affiliations:** 1 Research Department, Sidra Medicine, Doha, Qatar; 2 Division of Gastroenterology, Hepatology and Nutrition, Sidra Medicine, Doha, Qatar

**Keywords:** Crohn’s disease, Ulcerative colitis, Inflammatory Bowel disease, Markers, Anti-TNF, Infliximab, Adalimumab

## Abstract

Inflammatory bowel disease (IBD) is a chronic gastrointestinal condition characterized by severe gut inflammation, commonly presenting as Crohn’s disease, ulcerative colitis or categorized as IBD- unclassified. While various treatments have demonstrated efficacy in adult IBD patients, the advent of anti-TNF therapies has significantly revolutionized treatment outcomes and clinical management. These therapies have played a pivotal role in achieving clinical and endoscopic remission, promoting mucosal healing, averting disease progression, and diminishing the necessity for surgery. Nevertheless, not all patients exhibit positive responses to these therapies, and some may experience a loss of responsiveness over time. This review aims to present a comprehensive examination of predictive biomarkers for monitoring the therapeutic response to anti-TNF therapy in IBD patients. It will explore their limitations and clinical utilities, paving the way for a more personalized and effective therapeutic approach.

## Introduction

Inflammatory bowel disease (IBD) is a chronic, recurrent inflammatory disorder of the gastrointestinal (GI) tract, resulting in a wide range of clinical symptoms [1]. The global incidence of IBD is increasing, affecting even younger individuals and children, possibly due to various factors such as urbanization, westernization, dietary changes, increased antimicrobial exposure, and alterations in the host-microbial balance [2]. IBD is characterized by functional damage to the GI tract and intestinal epithelium, requiring lifelong medication [1]. Based on clinical symptoms, endoscopy imaging, and histology, IBD is broadly categorized into three major subtypes, Ulcerative Colitis (UC), which primarily affects the colon and Crohn’s Disease (CD) which affects multiple sites within the GI tract [3]. This third subtype, where histology assessments do not categorize patients into either Ulcerative Colitis (UC) or Crohn’s Disease (CD), is referred to as ‘‘Inflammatory Bowel Disease, type unclassified’’ or ‘‘Undetermined’’ (IBD-U) [2]. Diagnosing patients with IBD-U is challenging due to the ambiguous nature of their symptoms. They may display characteristics of both UC and CD, or present with atypical symptoms that do not fit into either category [4]. The disease follows a pattern of remission and relapse and often leads to complications and the need for surgical intervention in a majority of cases [5].

Advances in our understanding of the underlying immunopathogenic mechanisms of IBD have paved the way for the development of targeted therapies. The initial class of biological therapies approved for the treatment of IBD patients focused on inhibiting the pro-inflammatory cytokine tumor necrosis factor (TNF) in 1998 [6]. This biologic has revolutionized IBD treatment, markedly enhancing response rates and increasing the chances of remission among patients, and exerting a significant influence on the existing therapeutic algorithms [7]. Anti-TNF-alpha therapies, such as Infliximab and Adalimumab, have proven effective for moderate to severe CD cases [8]. Infliximab, the first biologic used for IBD treatment, is a genetically engineered chimeric immunoglobulin (Ig)G1 anti-human TNF agent [9]. It can activate complement and target cells expressing membrane-bound TNF-alpha, effectively downregulating inflammatory mechanisms throughout the mucosal layer [10, 11].

Anti-TNF therapies are generally well-tolerated and landmark studies like the ACCENT I and II trials have contributed significantly to our understanding of their effectiveness [12, 13], however, not all patients respond well to these biologics [14–16]. In addition, these agents carry risks of infection and malignancy, contributing to treatment costs [17]. Therefore, effective management of patients requires careful consideration of essential factors, including the potential for immunogenicity, the safety profile of treatments, and the optimal therapeutic duration. Over the past decade, numerous studies have identified a range of predictive biomarkers that hold the promise of delivering personalized and effective treatments for patients [8, 18]. This review will provide a comprehensive summary of findings derived from various studies in predicting response to anti-TNF therapies. Additionally, the review will highlight the limitations and clinical utilities of these predictive biomarkers in monitoring optimized patient outcomes and delivering personalized care.

### Loss of response to anti-TNF therapy

Over the last two decades, anti-TNF-alpha therapies have emerged as highly promising therapies for autoimmune and inflammatory conditions such as IBD, rheumatoid arthritis, psoriasis, and ankylosing spondylitis. However, a significant challenge lies in the fact that not all patients respond favorably to these therapies [19]. Approximately 30% of IBD patients do not respond to anti-TNF therapy (primary non-responders), and nearly half of those who initially benefit from these drugs experience a loss of clinical benefits within the first year, necessitating dose escalation or treatment alteration, referred to as “secondary loss of response” [20, 21]. Therefore predicting response to IBD therapy is crucial to avoid severe IBD-associated complications such as surgery and hospitalizations. Indeed these predictive biomarkers are instrumental in administering the appropriate treatment to the right patient at the right time. Moreover, considering that a significant number of IBD patients either develop intolerance or experience a loss of response to treatment over time, the ability to predict treatment response opens avenues for more personalized treatment options [22]. Recent studies have unveiled a wealth of potential markers, encompassing both genetic and non-genetic factors, that show promise as indicators for predicting responses to anti-TNF therapies.

#### Predictive genetic markers

Some genetic markers were shown to have a correlation with predictive primary response to the biological treatment in IBD patients. For example, a favorable clinical response was observed to be positively correlated with polymorphisms in genes like FCGR3A, TLR4, TNFRSF1A, IFNG, IL6, and IL1B. Conversely, variants of TLR2 and TLR9 showed a negative correlation with treatment response and are categorized as primary non-responder [23]. Although, most genetic predictive markers are related to cytokines/chemokines or their receptors and immunoglobulin receptors, including *TNF* gene, TNFRSF1B gene, *ATG16L1, and ATG16L2* gene, apoptosis genes, *NOD2/CARD15* genes, *IL23R* and *IL12* genes and Fc receptors related genes [24–26]. For example, genetic variants in TNF or TNFRSF1B gene and NFkB gene, may influence the level of TNF-alpha production or affect the binding affinity of TNF-alpha blockers to TNF-alpha receptor and, in turn, affect the primary response to anti-TNF (Infliximab, adalimumab, and golimumab) therapy in CD patients [27–29] (Table 1), or UC patients [30] (Table 2). Another polymorphism in NOD2/CARD15 gene has been linked to CD and may influence the response to anti-TNF-alpha therapy in individuals with this mutation, while polymorphisms in the IL23 receptor have been linked to the response to Infliximab treatment in UC patients [31]. In addition, when examining the Fas ligand gene, a CC genotype related to apoptosis has been positively associated with non-primary responsiveness to Infliximab. In contrast, individuals with a TC or TT genotype have been predictive of a positive response to anti-TNF therapy [27]. Moreover, investigations into the FCGR3A and ATG16L1 gene polymorphisms have revealed their impact on response to anti-TNF treatment. [25, 26, 32]. IBD patients with ATG16L1 T/T and C/T genotypes have demonstrated significantly higher CRP levels and a more favorable response to Adalimumab compared to those with the C/C genotype [24, 33]. Similarly, some specific genetic variations in genes like TNF-β and TNFRSF1B (rs1061624_A‐rs3397_T), in conjunction with a minor allele (A) polymorphism of the TNF gene (rs1800629), have been linked to the prediction of non-responsiveness to anti-TNF (Infliximab) therapy among patients with CD [27–29]. Conversely, a heterozygous genotype of IL12B—10,993 G > C (rs3212217) has shown a positive correlation with non-responsiveness to anti-TNF therapy in patients with UC [30, 34]. Hence, patients with these genetic profile are categorized as primary non-responders to anti-TNF therapy.

**Table 1:** Putative biomarkers for evaluating anti-TNF therapeutic efficacy in CD patients

Candidate gene	Anti-TNF therapy: CD patients	
	Genetic variants NR vs R	Expression NR vs R
• TNFSF4, TNFSF18	rs116724455[C/T]	–
• PLIN2, HAUS6	rs2228416 [T/C]	–
• LTF, CCR5, CCRL2	rs762787 [T/C]	–
• KLHL1	rs9572250 [G/A]	–
• PROX1, RPS6KC1	rs144256942 [G/A]	–
• RORB, TRPM6	rs523781[G/C]	–
• TLR2	rs3804099 [C/T]	–
• TLR2	rs11938228 [C/A]	–
• TLR2	rs1816702 [T/C]	–
• TLR2	rs4696480 [A/T]	–
• TLR4	rs5030728 [G/A]	–
• TLR9	rs352139 [G/A]	–
• NOD2	rs2066844 [C/T]	–
• NOD2	rs2066845 [G/C]	–
• NOD2	rs41450053[C/G]	–
• LY96	rs11465996 [G/C]	–
• IFNG	rs2430561 [A/T]	–
• IL17A	rs2275913 [A/G]	–
Fas ligand	843 CC/TT	–
Caspase-9	93 TT/CC	–
Mucosal transcripts • TNF-α	–	↑
• IL-17A	–	↑
• OSM	–	↑
• IL-7R^a^	–	↑
• miRNAs	–	↑
• TREM1	–	↓
Proteomics	–	↑
Genomic	–	↑

^a^A reduced mucosal transcript levels of IL-7R also observed in responders to immunosuppressive/corticosteroids, anti-TNF, or anti-a4b7 therapies in severe CDpatients. *TNF-α* tumour necrosis factor-α, IFN-γ: interferon-γ, IL-*17A* interleukin-17A, *miRNAs* MicroRNAs, *OSM* Oncostatin M, *IL-7R* interleukin-7 receptor, ↓: increase in expression, -: not known. A, adenine,*C*, cytosine; chr, chromosome, G, guanine; MAF, minor allele frequency; *NR* no response, *OR* odds ratio, *R* response, *T* thymine, *TNF* tumour necrosis factor, *CI* confidence interval, *IBD* inflammatory bowel disease, *TREM1* Triggering receptor expressed on myeloid cells 1

**Table 2:** Putative biomarkers for evaluating anti-TNF therapeutic efficacy in UC patients

Candidate gene	Anti-TNF therapy: UC patients	
	Genetic variants NR vs R	Expression NR vs R
• TLR2	rs3804099 [T/C]	–
• TLR2	rs11938228 [C/A]	–
• TLR2	rs1816702 [T/C]	–
• TLR2	rs4696480 [A/T]	–
• TLR4	rs5030728 [G/A]	–
• TLR9	rs352139 [G/A]	–
• LY96	rs11465996 [G/C]	–
• IFNG	rs2430561 [A/T]	–
• IL17A	rs2275913 [A/G]	–
Mucosal transcripts • TNF-α	–	↑
• IL-17A	–	↑
• IFN-γ	–	↑
• OSM	–	↑
• IL-7R^a^	–	↑
• miRNAs	–	↑
• TREM1	–	↓
HP	–	↑
CD177		↑
GPR84		↑
CST7		↑
S100A12		↑
ARG1		↑
ANXA3		↑
ANKRD22		↑
Genomic		↑

^a^A reduced mucosal transcript levels of IL-7R also observed in responders to immunosuppressive/corticosteroids, anti-TNF, or anti-a4b7 therapies in both severe UC patients. *TNF-α* tumour necrosis factor-α, *IFN-γ* interferon-γ, *IL-17A* interleukin-17A, *miRNAs* MicroRNAs, *OSM* Oncostatin M, *IL-7R* interleukin-7 receptor, ↓ increase in expression, -: not known. A, adenine, *C* cytosine, *chr* chromosome, *G* guanine, *MAF* minor allele frequency, *NR* no response, *OR* odds ratio, *R* response, *T* thymine, *TNF* tumour necrosis factor, *CI* confidence interval, *IBD* inflammatory bowel disease, *TREM1* Triggering receptor expressed on myeloid cells 1. HP- Haptoglobin, CD177-NB1 glycoprotein, GPR84-G-protein coupled receptor 84, CST7-Cystatin F, S100A12- S100 calcium-binding protein A12, ARG1-arginase 1, ANXA3-Annexin A3, ANKRD22- ankyrin repeat domain 22

#### Fecal markers

Although, fecal calprotectin and lactoferrin are commonly considered surrogate markers for assessing luminal disease activity [35]. These markers have been suggested as potential indicators of how individuals with CD [36] and UC [37] patients might respond to anti-TNF therapy [38]. However, the results from various studies present a mixed picture. In some instances, higher calprotectin levels were associated with a better response [39], while in others, the relationship was the opposite [40, 41]. Additionally, certain studies have failed to confirm any of these associations [42–44]. In summary, it appears that fecal calprotectin levels alone may not reliably predict an individual patient's response to anti-TNF therapy. While analyzing metabolic network reconstruction and metabolic profiles in fecal samples might offer insights into which patients with IBD are more likely to achieve clinical remission with anti-TNF therapy [45].

#### Immune markers

Monitoring changes in blood or mucosal parameters has proven valuable in evaluating the effectiveness of anti-TNF therapy. Effective anti-TNF therapy results in a decrease in TNF-α and interferon (IFN)-γ levels, signifying reduced inflammation at the mucosal level [41, 46]. In addition, Th17 signature cytokines, including IL-17A, IL-17B, IL-17D, and IL-17F, have also shown potential as candidate biomarkers for assessing the efficacy of anti-TNF therapy in patients with IBD [38]. They can modulate the expression of various cytokines and chemokines associated with autoimmunity and immune functions. In non-responders compared to responders, genes in the chemokine signaling pathway are down-regulated. Normally, high mucosal expression of IL7R, IL-6, sTNFR2, IL-1, IL-10, IL-8, and oncostatin M (OSM) signaling before treatment is associated with primary non-response to anti-TNF therapy in CD and UC patients [47–51]. Although these immune biomarkers might be considered as biomarker candidates for the evaluation of anti-TNF therapeutic efficacy in IBD patients, however, the clinical utilities remain a challenge, and further validation studies are required to confirm their potential as a biomarker. Currently, to the best of our knowledge no comparative studies assess the efficacy of anti-TNF treatments in IBD patients with normalized versus high TNF levels. Moreover, treatment decisions normally rely on disease activity evaluations based on clinical symptoms and endoscopic observations. It remains uncertain whether IBD patients with normalized mucosal TNF levels can benefit from anti-TNF therapy.

#### Protein markers

Proteomics can provide valuable insights into treatment responses to anti-TNF therapy, disease mechanisms, and individualized patient care; hence proteomics profiling is rapidly being explored to identify predictive biomarkers for monitoring or therapeutic response [52, 53]. For example, recent studies have identified multiple proteins markers (ANG1, ANG2, CRP, CEACAM1, SAA1, EMMPRIN, TGFA, MMP1, MMP2, MMP3, MMP9, IL-6, IL-7, sCD40L, PF4, complement C4-B, apolipoprotein A-I, apolipoprotein E, serotransferrin, beta-2-glycoprotein 1 and clusterin and VCAM1) in CD patients responding to Infliximab [53, 54]. Although proteomics has great potential for evaluating therapeutic responses, individual responses to therapy can be influenced by multiple factors beyond proteomics, such as genetics, lifestyle, microbiome, and environmental factors. Hence proteomics markers alone are not enough to predict the efficacy of biologics, an integration of proteomics data with other omics data as well as clinical information will provide better predictive universal markers for therapeutic response in IBD patients.

#### Microbial biomarkers

Although the etiology of IBD remains exclusive, the complex interaction of the gut microbial communities with immune cells can influence the disease severity and susceptibility to immune therapy in IBD patients [2]. The relationship between the gut microbiome and anti-TNF therapy is complex and not fully understood, however, research on the role of the microbiome in the context of anti-TNF-alpha therapy is continuously evolving. The gut microbiome, which consists of trillions of microorganisms residing in the gastrointestinal tract, plays a crucial role in immune modulation [55–58], intestinal inflammation [59], and response to immunomodulatory therapies [2, 60, 61], including anti-TNF-alpha drugs [62]. Emerging studies have suggested that certain microbial markers may be associated with treatment outcomes and response to anti-TNF-alpha therapy [62, 63]. For example; diverse microbes are generally associated with a healthier gut and patients with a more diverse gut microbiome respond better to anti-TNF-alpha therapy, while the presence of certain microbial species or groups of bacteria are associated with either a positive or negative response to anti-TNF-alpha therapy [63]. For example, a higher abundance of *Faecalibacterium prausnitzii, Ruminococcus bromii, Bifidobacterium ssp., Clostridium colinum*, *Eubacterium rectale,* and lower levels of *Streptococcus mitis* have have been linked to a better treatment response to anti-TNF therapy in IBD patients [64–66]. Moreover, increased levels of butyrate‐producing species (such as *Roseburia inulinivorans* and *Burkholderiales*) and a higher level of branched-chain amino acids are shown to be positively correlated with the clinical response to Vedolizumab [67]. In contrast, short-chain fatty acids producing bacteria such as *Lachnospiraceae* and *Ruminococcaceae* families, have been found to be associated with primary non-responders to the anti-TNF-α therapies [68]. In addition, the patients with gut microbial dysbiosis [69] or with additional fibrostenotic disease often exhibit no-response or poor response to anti-TNF therapy [70–73]. The balance between the two dominant microbial groups mainly *Prevotella* and *Bacteroides* in the gut has also been explored in relation to anti-TNF therapeutic response in patients [74, 75]. Higher levels of *Prevotella* relative to Bacteroides have been associated with better response to biologics [76]. Interestingly, a recent study by Caenepeel et al. (2024) investigated various combinations of clinical and microbial data to predict the efficacy of TNF-alpha (infliximab, adalimumab, and golimumab) in patients with both CD and UC [77]. Their model, integrating clinical parameters, stool features (moisture and calprotectin), and identification of microbial dysbiosis, achieved a 73.9% accuracy rate in predicting treatment outcomes with different biologicals. Notably, the study unveiled the significant microbiota-modulating effect of anti-TNF-α therapy, while vedolizumab appeared less effective in patients with dysbiotic microbiota. In addition, abundance of Fusobacterium also correlated with fecal calprotectin concentrations and postoperative relapse in patients with CD [78].

In addition to changes in the bacteriome, dysbiosis in the mycobiome also plays a crucial role in the pathophysiology of IBD and may influences responses to therapy. For instance, an increase in Basidiomycota and a decrease in Ascomycota, especially Saccharomyces cerevisiae, are associated with disease activity and biologic response [79]. Shifting Basidiomycota to Ascomycota ratios are observed during remission, hence indicating a potential marker for fungal dysbiosis [80]. Notably, the abundance of *Candida albicans* in IBD is reduced in patients who are primary responders to anti-TNF-alpha therapies [80]. While viruses are more abundant in the microbiome compared to bacteria, there is limited exploration of the inflammatory bowel disease (IBD) virome. Despite the bacteriome being a more accurate reflection of IBD activity, specific viruses such as those from the Retroviridae family are associated with CD [81]. However, their precise connection to the response to anti-TNF-alpha therapies remains unclear [82]. Although finding microbial biomarkers for clinical response to biological therapies seems to be a promising target, considering the diversity of changes in different populations and lack of statistical power among studies, it is important to note that research in this area is ongoing, and findings may not yet have fully translated into routine clinical practice.

#### Anti-drug-antibodies

**Fig. 1:**
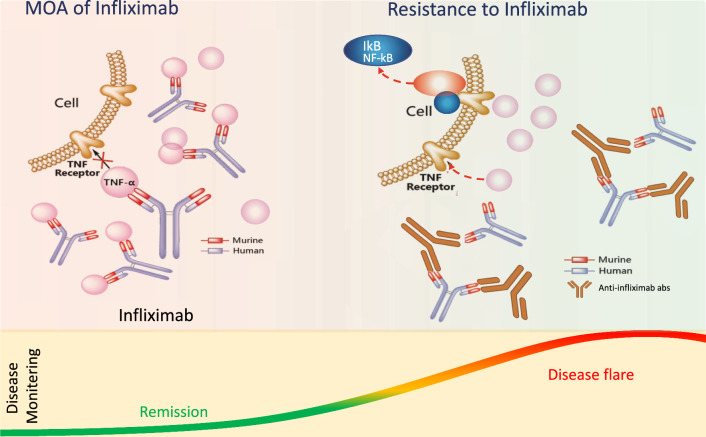
Molecular resistance mechanisms to anti-TNF therapy in IBD Patients. *MOA* mechanism of action

In some patients, anti-TNF therapy can stimulate the immune system to produce neutralizing anti-drug antibodies (ADAs) [83, 84]. This process is known as immunogenicity, which can be modulated by various factors and is a crucial consideration in IBD treatment especially with biological therapies. For example, certain genetic factors have been associated with an increased risk of ADA formation in CD patients undergoing anti-TNF therapy. These factors include HLA-DQA1*05, HLA-DRB1 alleles, and polymorphisms at the FCGR3A locus, which encodes the IgG Fc receptor IIIa [85, 86]. Monoclonal antibodies, particularly chimeric antibodies like Infliximab, can stimulate the production of ADA [87], which can lead to treatment inefficacy or secondary loss of response (Fig. 1), therefore, monitoring of serum ADAs and anti-TNF levels in case of > 3 μg/ml Infliximab therapy in IBD patients is an important parameter to optimum treatment response in IBD patients [88]. Although the presence of ADAs in patients is not permanent, it may disappear within a year after discontinuing the anti-TNF therapy [89]. In addition, some studies have also reported a link between poor efficacy of Infliximab therapy and anti-OmpC (*Escherichia coli* outer membrane porin) antibodies as well as anti-neutrophil cytoplasmic antibodies (pANCA) [90–92]. However, testing for pANCA positivity to predict non-response to Infliximab therapy showed a sensitivity of only 25% and a specificity of 85% resulting in the pANCA testing not commonly being employed in routine clinical practice for predicting therapy response [93].

#### Others

Loss of responsiveness to anti-TNF therapies in IBD might also be due to increased activity of matrix metalloproteinases (MMPs), which break down anti-TNF antibodies [94], and heightened clearance of TNF-anti-TNF antibody complexes through Fc receptor-mediated endocytosis, leading to proteolytic degradation [77] and contributing to secondary loss of response in IBD patients [95].

MicroRNAs (miRNAs) are small non-coding RNAs, and play an important role in pro-inflammatory cytokine production and the inflammatory processes in IBD patients [96]. In recent years, circulating and fecal miRNAs have emerged as potential novel biomarkers for predicting therapeutic responses in IBD patients [97]. For example, Batra et al. demonstrated significant changes in the expression of seven miRNAs after treatment in responders compared to non-responders, within a small cohort of pediatric IBD patients receiving anti-TNF therapies [98]. However, another study focusing on miRNA polymorphisms and their association with anti-TNF treatment response in CD did not find any correlations between identified miRNA polymorphisms (miR-146 rs2910164, miR-196a rs11614913, miR-221 rs113054794, and miR-224 rs188519172) and patients' responses to anti-TNF mAbs in CD patients [99]. Therefore, further studies are required to fully investigate and validate the utility of miRNAs as predictive markers for treatment outcomes in IBD.

Previous surgery or exposure to anti-TNF therapy in IBD patients has been associated with an increased risk of both primary non-responders and secondary loss of response in subsequent anti-TNF therapies [100–102]. The effectiveness of a second anti-TNF treatment appears to be influenced by the reason for switching, with higher remission rates observed in patients who discontinued anti-TNF therapy due to intolerance (61%) compared to those with secondary (45%) or primary failure (30%) [103]. Additionally, concomitant immunomodulator therapy, as shown in the SONIC trial for CD patients [104] and the SUCCESS trial for UC patients [105], has been demonstrated to enhance corticosteroid-free remission rates when combined with infliximab. Although, these data suggest that non-genetic, environmental factors can influence the response to anti-TNF therapies in IBD patients, they do not pinpoint any specific markers or concrete indicators.

## Future directions

In summary, the discovery of anti-TNF therapies has resulted in remarkable changes in the treatment approach of IBD, shifting from gradual attainment of symptomatic clinical remission to achieving sustained remission. This transformation has enabled the realization of short- and long-term clinical and endoscopic remission, a goal once considered unattainable [53, 106]. As a result, anti-TNF therapies occupy a central position within the current IBD treatment paradigm, and they can be further enhanced through strategies such as continuous monitoring of disease progression and/or response to therapy [107].

Although the use of laboratory biomarkers shows potential for enhancing the evaluation and customization of anti-TNF therapy for IBD, the search of predictive biomarkers is still in its nascent phase. Significant challenges remain to be addressed.. Many of the identified biomarkers primarily indicate a generalized inflammation and lack specificity for IBD. In reality, the response to this therapy is influenced by a variety of factors, including disease-related characteristics, biochemical markers, genetics, microbial composition, metabolic factors, and local mucosal conditions. As a consequence, this could be the underlying reason why none of the identified biomarkers have been incorporated into routine clinical practice as definitive tools for enabling personalized treatment approaches. The recent model reported by Caenepeel et al. which integrated a combination of clinical data, stool characteristics (microbial load, moisture content, and calprotectin concentration), and fecal microbiota, enabled the prediction of treatment response to biological therapies [77]. However, it still classified more than half of IBD patients as refractory to all anti-inflammatory therapies studied [77]. To address this, future efforts should focus on robustly validating biomarkers through well-designed studies involving a significant number of IBD patients.

Nevertheless, despite their pivotal role in the current IBD treatment paradigm, certain aspects of anti-TNF use still pose unanswered questions. These include considerations about the timing, dosing, monitoring, and addressing issues related to loss of treatment response, such as identifying risk factors, biomarkers, mechanistic insights, and devising strategies for both prevention and management. More efforts should be focused on leveraging advanced multi-Omics and computational techniques to assess gut microbiota and immune signatures of IBD patients before and during treatment. These approaches can help pinpoint dysbiotic microbiota and mucosal signatures, enabling tailored treatment plans involving microbial consortia and anti-inflammatory therapies. Several promising novel treatment strategies are in clinical trials for IBD. The ultimate goal should be to establish a comprehensive profile including genetics, cytokines, inflammatory markers, and microbiota at the time of diagnosis, allowing clinicians to tailor specific treatments to individual patients—a true precision medicine approach for IBD. Integration of multi-omic data may offer insights into treatment response patterns, yet adoption in clinical practice is hindered by resource scarcity and lack of standardized methodologies. We feel IBD organizations and policy mackers can play a crucial role in formulating clear methodologies, secure funding for inovative multi-omic approaches into research as well as for easily implementable clinical tools, leading to effective clinical solutions for IBD in the near future.

## Data Availability

All available data is presented in the submitted work.
